# Vitamin C enhances epigenetic modifications induced by 5-azacytidine and cell cycle arrest in the hepatocellular carcinoma cell lines HLE and Huh7

**DOI:** 10.1186/s13148-016-0213-6

**Published:** 2016-04-30

**Authors:** Sahar Olsadat Sajadian, Chaturvedula Tripura, Fazel Sahraneshin Samani, Marc Ruoss, Steven Dooley, Hossein Baharvand, Andreas K. Nussler

**Affiliations:** Eberhard Karls University Tuebingen, BG Trauma Clinic, SWI, Schnarrenbergstraße 95, 72076 Tuebingen, Germany; CSIR - Centre for Cellular and Molecular Biology, Uppal Road, Habsiguda, Hyderabad India; Department of Stem Cells and Developmental Biology, Cell Science Research Center, Royan Institute for Stem Cell Biology and Technology, ACECR, Tehran, Iran; Department of Medicine II, Section Molecular Hepatology, Medical Faculty Mannheim, Heidelberg University, Heidelberg, Germany

**Keywords:** 5-Azacytidine, Vitamin C, TETs, 5hmC, Snail, GADD45B, EMT/MET

## Abstract

**Background:**

5-Azacytidine (5-AZA), a DNA methyl transferase inhibitor, is a clinically used epigenetic drug for cancer therapy. Recently, we have shown that 5-AZA upregulates ten-eleven translocation (TET) protein expression in hepatocellular carcinoma (HCC) cells, which induce active demethylation. Vitamin C facilitates TET activity and enhances active demethylation. The aim of this study is to investigate whether vitamin C is able to enhance the effect of 5-AZA on active demethylation and to evaluate its consequence in HCC cell lines.

**Methods:**

HCC cell lines (Huh7 and HLE) were treated with 5-AZA and vitamin C. After 48 h of treatment, viability (resazurin conversion), toxicity (lactose dehydrogenase (LDH) release), and proliferation ((proliferating cell nuclear antigen (PCNA)) of single- and combined-treated cells were assessed. The effect of the treatment on 5-hydroxymethylcytosine (5hmC) intensity (immunofluorescence (IF) staining), TET, Snail, GADD45B, and P21 mRNA (real-time PCR) and protein expression (Western blot) were investigated.

**Results:**

Our results indicated that vitamin C enhances the anti-proliferative and apoptotic effect of 5-AZA in HCC cell lines. By further analyzing the events leading to cell cycle arrest, we have shown for the first time in HCC that the combination of 5-AZA and vitamin C leads to an enhanced downregulation of Snail expression, a key transcription factor governing epithelial-mesenchymal transition (EMT) process, and cell cycle arrest.

**Conclusions:**

We conclude that when combined with 5-AZA, vitamin C enhances TET activity in HCC cells, leading to induction of active demethylation. An increase in P21 expression as a consequence of downregulation of Snail accompanied by the induction of GADD45B expression is the main mechanism leading to cell cycle arrest in HCCs.

## Background

Hepatocellular carcinoma (HCC) is the most common adult liver malignancy that shows relatively poor prognosis and rapid progression [1, 2]. It is now established that tumor cells undergo various epigenetic modifications, particularly DNA hypermethylation, that could lead to an imbalance in regulation of pro- and anti-apoptotic genes, which is attributed as one of the important factors in the progression and treatment of cancer [1, 3].

Recently, demethylation of 5-methylcytosine (5mC) to 5-hydroxymethyl cytosine (5hmC) was shown to be mediated by ten-eleven translocation (TET) proteins [4–6]. Since hypermethylation of promoters of tumor suppressor genes has been identified as one of the principal factors supporting cancer development, demethylation agents have now become the main focus of molecular-targeted therapeutics. Various in vitro studies have shown that 5-azacytidine (5-AZA), a potent DNA methyl transferase inhibitor (DNMTi), leads to re-expression of silenced genes and altered expression of genes involved in tumor suppression [7]. However, a very few studies have been published to understand the mode of action of 5-AZA. Recently, our lab clearly demonstrated that 5-AZA modulates the expression of genes through induction of TET2 and TET3, improving 5hmC generation in HCC and inhibiting cells proliferation [8].

All TET proteins contain a catalytic domain which binds to Fe^2+^ and 2-oxoglutarate to mediate oxidation of 5mC to 5hmC in DNA [4]. Vitamin C plays a central role in the conversion of 5mC to 5hmC by enhancing the catalytic activity of TET dioxygenases [9, 10]. In addition, recent reports point to the important role of vitamin C in modulating mesenchymal-epithelial transition (MET) by regulating TET1 [11] and also in inducing cell death via epigenetic modification in melanoma cells [12]. Therefore, vitamin C might be an important factor in reducing the risk of promoter hypermethylation supporting the maintenance of the 5hmC state and thus might play a major role in the epigenetic regulation [9, 11, 13, 14].

Transition from epithelial to mesenchymal (EMT) state is partially mediated by Snail expression, which is considered as a hallmark of cancer progression [15–17]. Transient Snail expression suppresses the epithelial marker E-cadherin whose downregulation is directly associated with tumor invasion and metastasis in HCC [18–20]. In addition to the control of EMT, Snail as a major transcriptional repressor is also involved in the regulation of cell cycle progression and in conferring resistance to programmed cell death [21].

Several reports have shown that 5-AZA induces cell cycle arrest and cell death in tumor cells [22, 23] by altering the epigenetic state, but the details of 5-AZA-induced cell cycle arrest in HCC are not completely understood. Accumulating evidences also point to the significance of epigenetics in regulating EMT in cancer [reviewed in [24]]. A recent report shows MET in trophoblast cells by induction of epithelial markers when treated with a 5-AZA analog [25]. However, the effect of 5-AZA on Snail expression and the downstream pathways is not yet known. Hence, we have probed into the changes in gene expression of Snail which could possibly link EMT/MET and cell cycle arrest mediated by 5-AZA. Furthermore, we have also questioned if vitamin C as an epigenetic modifier could enhance the effect of 5-AZA in inducing cell death which probably could be explored as a possible combination therapy in HCC.

Our results have shown that 5-AZA induces cell cycle arrest by downregulating Snail and upregulating Gadd45 which was further enhanced by vitamin C.

## Methods

### Cell culture reagents, antibodies, and chemicals

DMEM medium and cell culture supplements were purchased from Sigma (Steinheim, Germany). Cell culture plastics, phosphate-buffered saline (PBS), and fetal calf serum (FCS) were purchased from PAA Laboratories GmbH (Pasching, Austria). DNase I (RNase-free) and First Strand cDNA Synthesis Kit were purchased from Fermentas (Ontario, Canada). 5-AZA and L-ascorbic acid 2-phosphate were obtained from Sigma-Aldrich (Steinheim, Germany). All other chemical compounds were purchased from Carl Roth (Karlsruhe, Germany). 5-hmC (39769) mouse mAB was purchased from Active Motif (Carlsbad, CA, USA). Proliferating cell nuclear antigen (PCNA) (ab92552) rabbit mAB was obtained from Abcam (Cambridge, UK). Corresponding secondary antibodies goat anti-rabbit Alexa 555 and goat anti-mouse 488 were acquired from Invitrogen (Carlsbad, CA, USA). Anti TET2 (SAB3500711), anti TET3 (SAB2700682), and anti glyceraldehyde 3-phosphate dehydrogenase (GAPDH) (G9545) antibodies were used from Sigma (Munich, Germany). GADD45B (SC33172) rabbit mAB was from Santa Cruz, and Snail (3879) rabbit mAB, E-cadherin (14472) mouse mAB, p21 (2947) rabbit mAB, cyclin-B1 (4138) rabbit mAB, and HRP-linked anti-mouse and anti-rabbit IgG secondary antibody were purchased from Cell Signaling (Beverly, MA, USA).

### Cell culture and treatment

HLE and Huh7 cell lines were purchased from ATCC and cultured as published previously [8]. The HCC cell lines were plated onto 6-, 24-, or 96-well plates. Twenty-four hours after plating, the cells were incubated with 10 μM of 5-AZA and 1 mM of vitamin C (L-ascorbic acid 2-phosphate) for 48 h. 5-AZA and vitamin C were administrated at the same time. The absence of mycoplasma contamination was regularly tested using a commercially available test (Venor®GeMtest, Minerva Biolabs GmbH, Berlin, Germany).

### Immunofluorescence staining

Immunostaining of 5hmC and PCNA was performed using the published method [26]. Briefly, the cells were plated onto cover slips and treated according to the experimental setup. After 48 h, the cells were fixed with 4 % paraformaldehyde solution for 15 min at RT and then washed with PBS. For permeabilization, the cells were incubated with 0.5 % Triton X-100 in PBS for 15 min at RT. To denature the DNA, the cells were incubated with 4 M HCl for 15 min at RT, rinsed with distilled water, and placed in 100 mM Tris-HCl (pH 8.5) for 10 min. After washing with PBS, non-specific binding sites were blocked with blocking buffer (10 % FCS, 0.1 % Tween-20 in PBS) for 1 h at RT. Then, the cells were incubated with primary antibodies such as anti-5hmC rabbit polyclonal IgG (Active Motif, CA, USA) or anti-PCNA Rabbit mAB (Abcam, Cambridge, UK) at 1:1000 and 1:200, respectively, in PBS solution containing 1 % FCS and 0.1 % Tween-20 overnight at 4 °C. After washing with PBS, the cells were incubated with secondary antibody solution (ALEXA-Fluor antibodies, Invitrogen, NY, USA), diluted 1:400 in PBS solution containing 1 % FCS and 0.1 % Tween-20 for 1 h at RT. Nuclei were counterstained by incubation with Hoechst 33342 solution (2 μg/ml in PBS) for 10 min at RT. After a final washing step with PBS, the stained cells were mounted with a mounting medium (Fluoromount G, Southern Biotech, NJ, USA). Images of the staining were taken with an EVOS fluorescence microscope (AMG, Life technologies, MA, USA) processed and analyzed with Image J 1.45s software (NIH, USA) [27].

### Real-time RT-PCR for detection of mRNA expression

Real-time PCR was performed as described previously [8]. Briefly, for the mRNA expression studies, total RNA was extracted using TriFast reagent (Peqlab, Erlangen, Germany); 2–3 μg of the total RNA was digested with DNase I in order to eliminate the contaminating genomic DNA according to the manufacturer’s instructions (Thermo Scientifics, CA, USA). Complementary DNA (cDNA) was synthesized by First Strand cDNA Synthesis Kit (Thermo Scientifics, CA, USA). For quantitative real-time PCR (qRT-PCR), 40 ng of template cDNA was used for the expression level of each target gene (primer sequences are listed in Table 1) using SYBR Green qPCR (Thermo scientific, Waltham, MA, USA) and the Step One Plus® Real-Time PCR System Kit (Life technologies, Carlsbad, CA, USA). All genes examined were normalized to a housekeeping gene encoding GAPDH. Relative expression values were calculated from Ct values using the ΔΔ_CT_ method with untreated cells as a control. Fold induction was calculated according to the formula 2^(Rt − Et)^/2^(Rn − En)^ [28]. PCRs were performed as follows: denaturation for 10 min at 95 °C, amplification with 40 cycles and 15 s at 95 °C, 40 s at 60 °C, and 15 s at 72 °C (Step One Plus™ Real-Time PCR System, Life technologies, CA, USA). The primer sequences are listed in Table 1. Each sample was set up in triplicates, and the experiment was repeated at least twice. Statistical significance of difference in target genes expression level between different treatment was assessed by *t* test, at *α* = 0.05.

**Table 1:** Primer sequences for real-time PCR

	Gene bank ID	Forward primer	Reverse primer	Product length (bp)
TET1	NM_030625.2	TCTGTTGTTGTGCCTCTGGA	GCCTTTAAAACTTTGGGCTTC	77
TET2	NM_001127208.2	GAGACGCTGAGGAAATACGG	TGGTGCCATAAGAGTGGACA	258
TET3	NM_001287491.1	CCCACAAGGACCAGCATAAC	CCATCTTGTACAGGGGGAGA	129
SNAIL	NM_005985.3	ACCACTATGCCGCGCTCTT	′GGTCGTAGGGCTGCTGGAA	115
E-cadherin	NM_004360.3	GTCAGTTCAGACTCCAGCCC	AAATTCACTCTGCCCAGGACG	254
GAPDH	NM_002046.4	TGCACCACCACTGCTTAGC	GGCATGGACTGTGGTCATGAG	87
P21	NM_001291549.1	GTCACTGTCTTGTACCCTTGTG	CGGCGTTTGGAGTGGTAGAAA	221
Gadd45	NM_015675.3	GTGTACGAGTCGGCCAAGTT	GTCACAGCAGAAGGACTGG-	135

### Resazurin conversion, LDH leakage, and FACS measurement

To investigate the impact of 5-AZA together with vitamin C on cell proliferation, the cells were incubated with different doses of 5-AZA and vitamin C at different time points. Cell viability (mitochondrial activity) was determined by resazurin conversion as described previously [8]. Briefly, 1/10 volume of the resazurin stock solution (0.025 % in DPBS) was added to the cells. After 30-min incubation at 37 °C, fluorescence was measured (Ex/Em = 544/590 nm) and corrected to background control (solvent mixture without cells) on a FLUOstar Omega microplate reader (BMG Labtech, Germany). Viability is given as percentage of control (untreated cells).

Lactose dehydrogenase (LDH), released into cell culture media as index of cell death, was measured using an LDH Assay Kit from Thermo Scientific (Rockford, USA) according to the manufacturer’s protocol. LDH released into the media was expressed as the percentage of the total cellular LDH per well measured after cells had been lysed with lysis buffer provided with the kit.

Cell cycle analysis and quantification of hypodiploid cells was determined by flow cytometry (FC). For FC analysis, 1 × 10^6^ HCC cells per well were seeded in 6-well plates and treated with different concentrations of 5-AZA and vitamin C. After 48 h, the cells were fixed in 70 % of ethanol for at least 1 h and stained in a hypotonic solution with 100 μg/ml of RNase and 50 μg/ml propidium iodide (PI) for 15 min in RT [29]. Distribution of cells in the different phases of the cell cycle based on the differences in DNA contents was determined using a flow cytometer (BD Biosciences, Heidelberg, Germany). Data were analyzed using Modfit software.

### Western blot

The treated and non-treated control cells were harvested after 48 h and lysed in ice-cold RIPA lysis buffer (50 mM Tris; 250 mM NaCl; 2 % Nonidet-P40; 2.5 mM EDTA; 0.1 % SDS; 0.5 % DOC; complete protease inhibitor; 1.0 % phosphatase inhibitor; pH 7.2). Protein concentration was determined by micro-Lowry. Thirty micrograms of total protein was separated by SDS-PAGE and transferred to nitrocellulose membranes (Roth, Karlsruhe, Germany). The membranes were blocked by 5 % blocking buffer (milk powder in Tris-buffered saline Tween (TBST)) for 1 h and incubated overnight with Snail, GADD48B, P21, cyclin B1, E-cadherin, TET2, TET3, and GAPDH mouse/rabbit polyclonal primary antibodies at 4 °C. The following day, the membranes were incubated with the corresponding HRP-labeled secondary antibodies for 1 h at RT. Chemiluminescent signals were detected with the ChemoCam (INTAS, Göttingen, Germany).

### Protein depletion by siRNAs

Depletion of TET2 and TET3 was achieved by small interfering RNA (siRNA) approach as reported previously [8]. Briefly, the HLE and Huh7 cells were transfected with TET2 and TET3 siRNAs, subsequently the transfected cells were treated with 5-AZA and vitamin C and harvested after 48 h along with appropriate negative controls that were transfected with scrambled siRNA. RNA isolated from treated knock-downs (KDs) and treated and untreated siRNA controls were used for gene expression analysis.

### Statistical analysis

Statistical significance of differences between the individual treatments was evaluated by one-way ANOVA Tukey’s test (Prism 5.01, GraphPad Software, San Diego, USA). Data are means ± SEM of three independent experiments. All statistical comparisons were performed two-sided in the sense of an exploratory data analysis using 0.05 (*), 0.01 (**), and 0.001 (***) level of significance.

## Results

### Vitamin C enhances the cytotoxic effects of 5-AZA and induces cell death

**Fig. 1:**
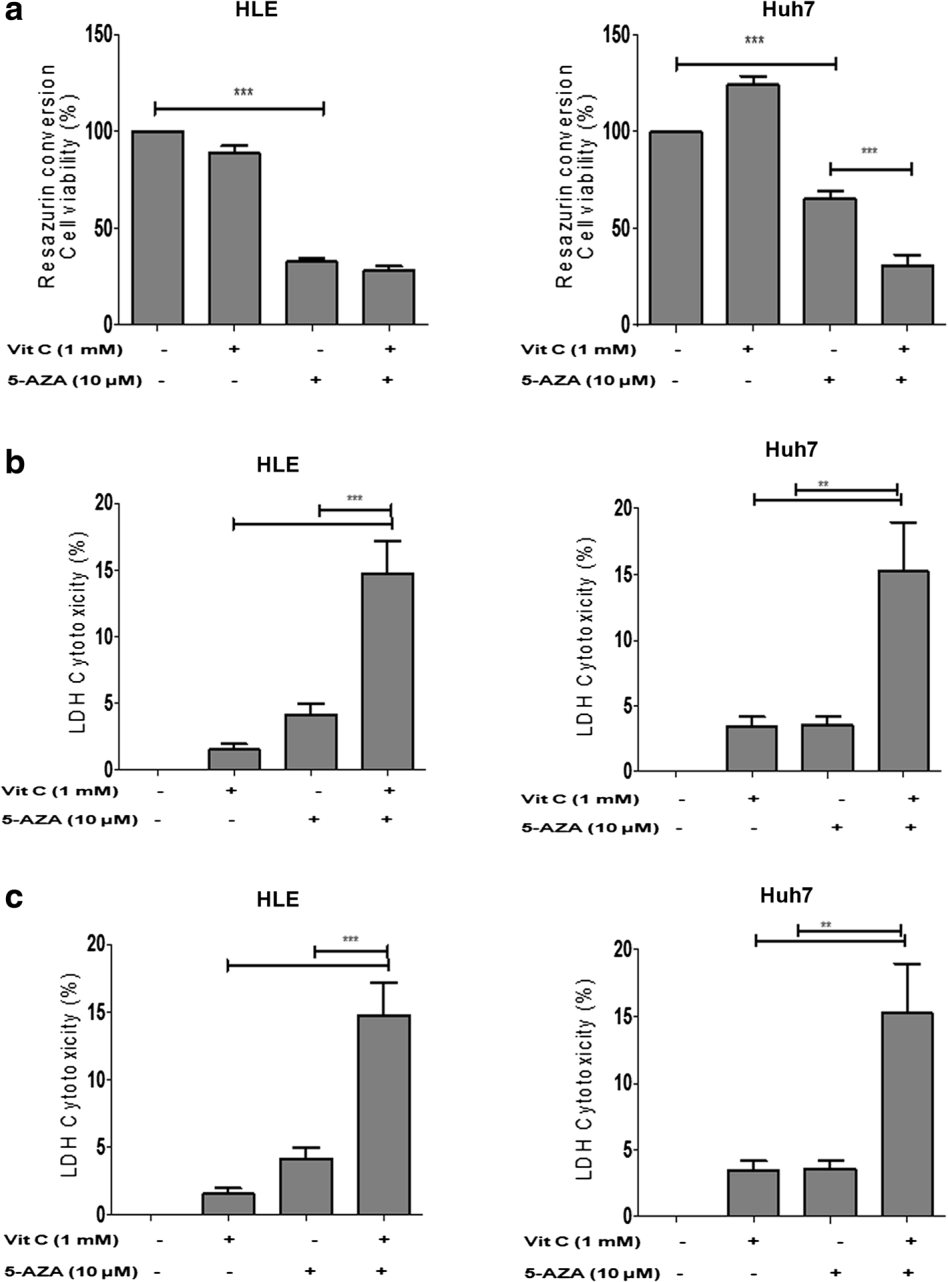
Vitamin C enhances the cytotoxic effects of 5-AZA in inducing cell death in HCC. **a** Changes in the resazurin conversion indicating the cell viability in HLE and Huh7 cells treated with vitamin C, 5-AZA, and 5-AZA + vitamin C compared to the untreated controls after 48 h. **b** Changes in the LDH release by HLE and Huh7 with the various treatments compared to the untreated control. **c** Flow cytometric measurement of sub2N population in HLE and Huh7 treated with vitamin C, 5-AZA, and 5-AZA + vitamin C. All the data are the average of the experiments (*N* = 3). Statistical significance was tested using one-way ANOVA (non-parametric) Tukey’s test. *p ≤ 0.05; **p ≤ 0.01; ***p ≤ 0.01. *Error bars* represent the standard deviation

The viability of the treated HCC cells was assessed as a function of mitochondrial activity by resazurin conversion assay and compared to the non-treated control cells. A reduced mitochondrial activity, reflecting a decrease in cell viability, was observed in the HLE and Huh7 cells treated with 5-AZA both with and without vitamin C, whereas vitamin C alone did not compromise the mitochondrial function after 48 h (Fig. 1a). In Huh7, cell viability was further reduced when 5-AZA was supplemented with vitamin C (approximately 30 %) (Fig. 1a).

In both, HLE and Huh7, inhibition of proliferation was paralleled by increased intracellular LDH enzyme release, indicating a leakage of intracellular contents by a compromise on the membrane integrity and induction of cell damage after 48 h of treatment (Fig. 1b). While a very low release of LDH was observed with 5-AZA and vitamin C individually, the combination of 5-AZA and vitamin C showed a high rate of cytotoxicity in both cell lines.

Further, flow cytometry analysis of the sub2N population as a measure of cell death revealed that the combination of 5-AZA and vitamin C induced a higher number of cells in the sub2N in HLE than in solely 5-AZA treated cells (Fig. 1c). In Huh7, a significant increase in the sub2N population was observed in cells treated with 5-AZA + vitamin C, with a slight increase in LDH compared to the 5-AZA single-treated cells (Fig. 1c).

### Inhibition of cell proliferation and induction of cell cycle arrest enhanced by the combined treatment of 5-AZA and vitamin C

**Fig. 2:**
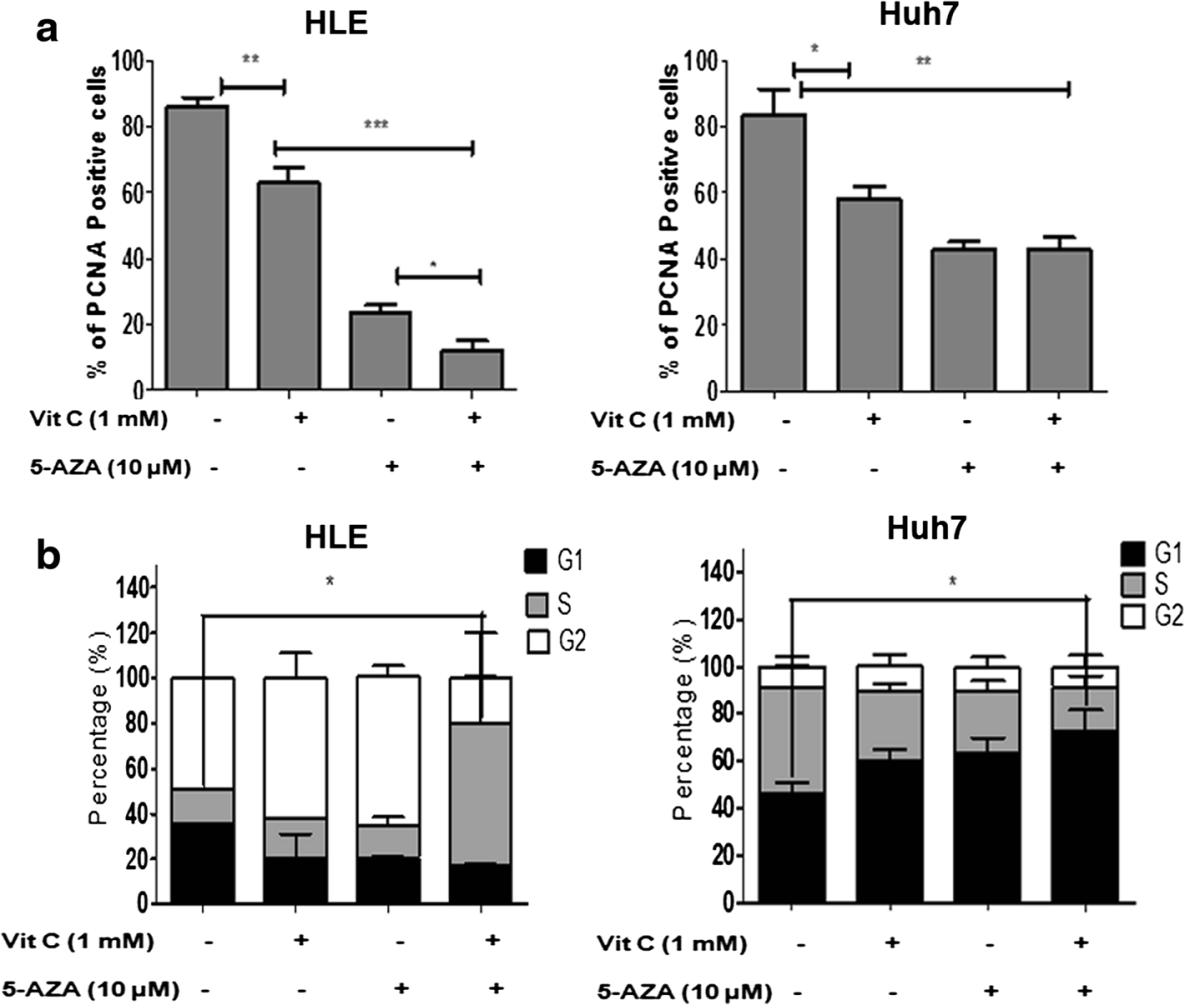
5-AZA and vitamin C inhibit cell proliferation and induce cell cycle arrest in HCC. **a** PCNA nuclear staining of HCC cell lines, HLE and Huh7, treated with vitamin C, 5-AZA, and 5-AZA + vitamin C for 48 h. *Graphs* represent the calculation of the percentage of PCNA-positive cells as an indicator of inhibition of cell proliferation in HLE and Huh7. **b** Cell cycle analysis indicating the stage of cell cycle arrest in HLE and Huh7. All the data are the average of the experiments (*N* = 3). Statistical significance was tested using one-way ANOVA (non-parametric) Tukey’s test. *p ≤ 0.05; **p ≤ 0.01; ***p ≤ 0.01. *Error bars* represent the standard deviation

To confirm the anti-proliferative effect of 5-AZA and vitamin C, expression of proliferation cell nuclear antigen (PCNA) was investigated by immunofluorescence staining (Fig. 2a). In comparison with the untreated control, inhibition of cell proliferation was observed in the HLE and Huh7 cells treated with vitamin C (Fig. 2a). In HLE, 5-AZA treatment induced a significantly higher inhibition, which was further enhanced with the combination treatment of 5-AZA + vitamin C. Similarly, in Huh7, a significant inhibition of proliferation was observed with both 5-AZA and the combination of 5-AZA + vitamin C (Fig. 2a).

Next, we determined by flow cytometry the phase of the cell cycle where the observed growth inhibition in both cell lines occurred. Cell cycle distribution analysis of the HLE cells treated with 5-AZA and vitamin C individually indicated an increase in the population of cells in G2 phase. However, a stronger increase in the S phase of the cell cycle was noted in cells treated with a combination of 5-AZA + vitamin C (Fig. 2b). In Huh7, we observed an increase in the population of cells in the G1 phase of the cell cycle with 5-AZA and vitamin C treatment. However, the number of cells in the G1 phase was highest with the combination treatment of 5-AZA and vitamin C (Fig. 2b).

### Vitamin C improves the efficacy of 5-AZA in TET-dependent active demethylation in HCC cell lines

**Fig. 3:**
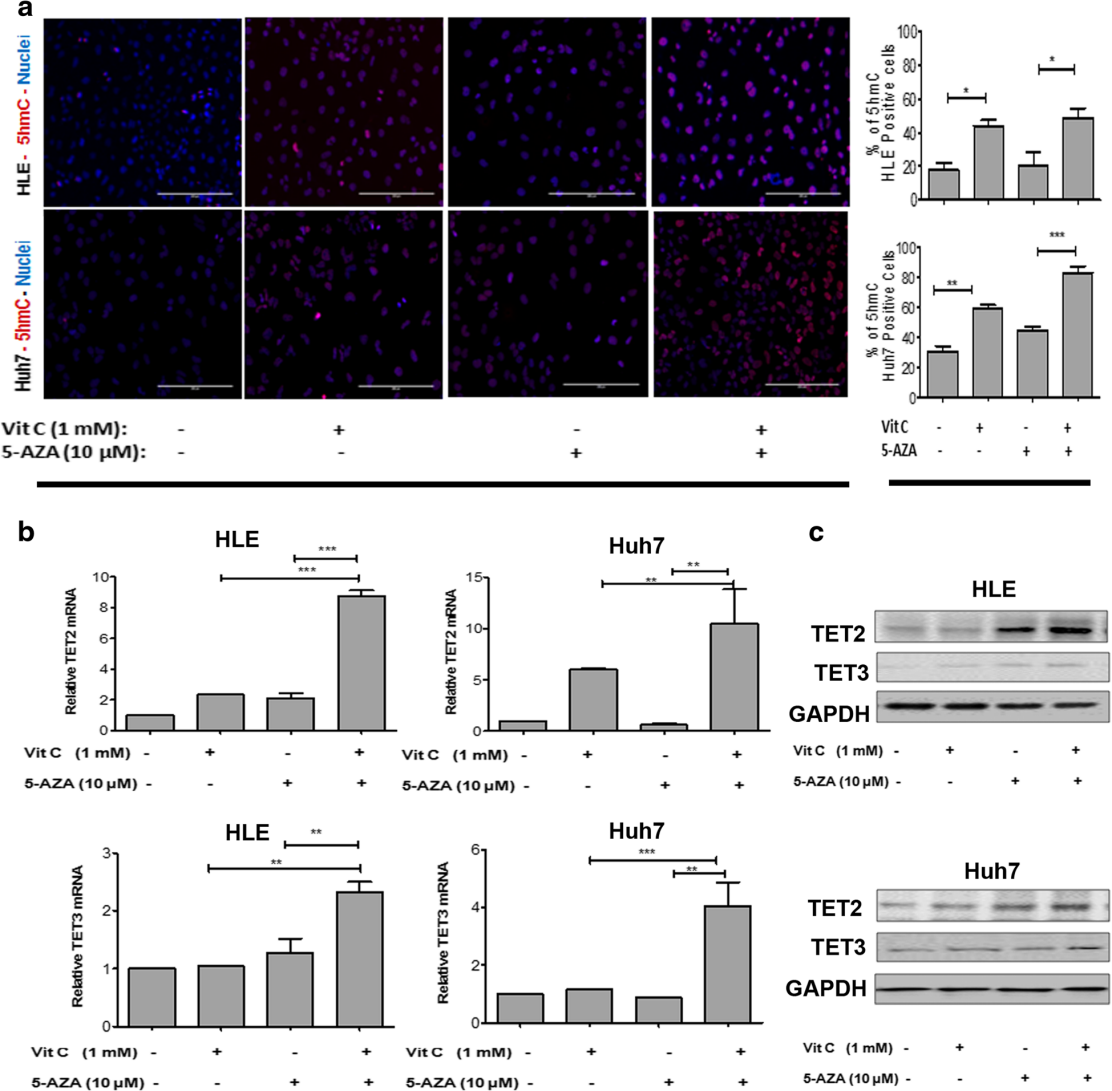
Vitamin C enhances the efficacy of 5-AZA in inducing active demethylation and generation of 5hmc by induction of TET expression in HCC. **a** 5hmC nuclear staining of HCC cell lines, HLE and Huh7, treated with vitamin C, 5-AZA, and 5-AZA + vitamin C. 5hmC-positive cells are seen in *red*, Hoechst is seen in *blue. White line* represents the scale bar (20 μm). *Graphs* show the calculation of the percentage of 5hmC-positive cells as an indicator of active demethylation in HLE and Huh7. **b** Quantitative changes in the mRNA expression of TET2 and TET3 in HLE and Huh7 cells with the various treatments. Data was normalized using GAPDH expression as a reference control. **c** Western blot results show changes of TET2 and TET3 protein expression in HLE and Huh7 cells with the various treatments. Data was normalized using GAPDH expression as a reference control. All the data are the average of the experiments (*N* = 3). Statistical significance was tested using one-way ANOVA (non-parametric) Tukey’s test. *p ≤ 0.05; **p ≤ 0.01; ***p ≤ 0.01. *Error bars* represent the standard deviation

In order to further evaluate the changes in the expression of genes which could have led to the cell cycle arrest, we first studied if the combination of 5-AZA and vitamin C induces any epigenetic changes in HCC cells. Since 5-AZA and vitamin C are both known to induce active demethylation which reflects changes in the 5hmC status [8, 11, 13, 14], we investigated the 5hmC content of the HCC cell lines treated with 5-AZA, vitamin C, and the combination of both after 48 h. Immunofluorescence (IF) staining of 5hmC indicated the presence of a significantly high percentage of 5hmc-positive cells with the combined treatment as compared to each single treatment in both HLE and Huh7 (Fig. 3a). The cells treated with vitamin C alone also showed an increase in 5hmC as compared to 5-AZA treatment or the untreated control, underlining the important role of vitamin C in active demethylation.

To investigate whether the effect of this increase in 5hmC intensity after treatment was correlated with changes in TET2 and TET3, the mRNA level of TET2 and TET3 was determined by real-time PCR. The cells treated with the combination of 5-AZA and vitamin C demonstrated a significantly increased expression of TET2 and TET3 as compared to the individually treated and non-treated controls in both HLE and Huh7 (Fig. 3b). In the Huh7 cells, vitamin C alone enhanced the expression of TET2 and TET3 while 10 μM of 5-AZA could not induce a significant increase in the expression of TET2 and TET3 (Fig. 3b). These data indicate the possibility that vitamin C when combined together with 5-AZA could influence the conversion of 5mC to 5hmC by inducing TET2 and TET3 expression. Our Western blot data also confirmed the increase of TET2 and TET3 after stimulation with 5-AZA and vitamin C (Fig. 3c).

### Induction of active demethylation by 5-AZA and vitamin C leads to downregulation of Snail and activation of GADD45B

Snail is a transcription factor regulated by methylation and has an important role in mediating EMT and in inducing tumorigenesis [21, 30]. Therefore, we first evaluated the effect of 5-AZA and vitamin C on Snail expression.

**Fig. 4:**
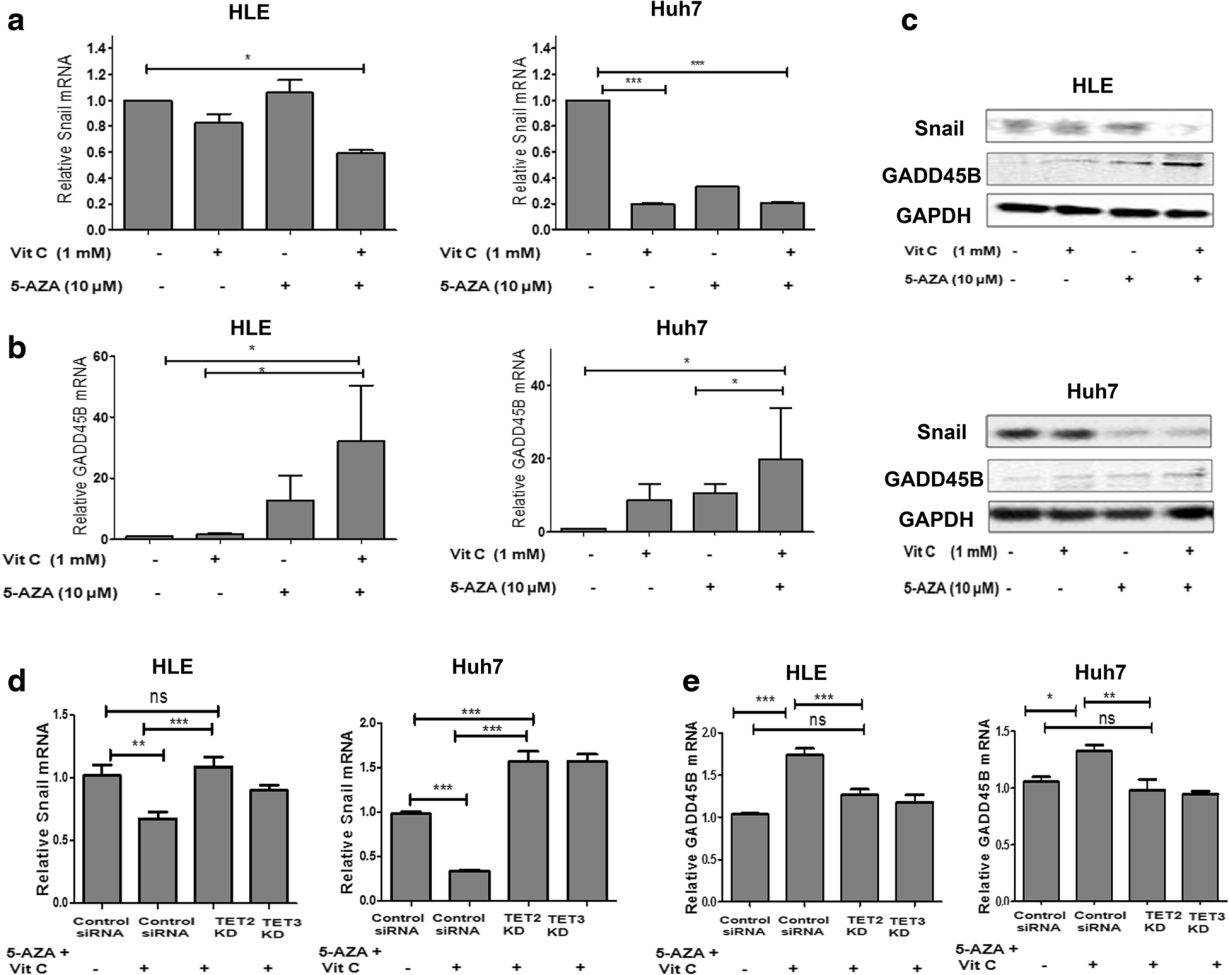
Vitamin C enhances the downregulation of Snail and upregulation of GADD45B expression induced by 5-AZA in HCC. **a** Quantitative changes in Snail mRNA in HLE and Huh7 treated with vitamin C, 5-AZA, and 5-AZA + vitamin C compared to the untreated control after 48 h. Data was normalized using GAPDH expression as a reference control. **b** Quantitative changes in GADD45B mRNA in HLE and Huh7 with the various treatments after 48 h. Data was normalized using GAPDH expression as a reference control. **c** Western blot analysis for changes in Snail and GADD45B proteins in HLE and Huh7 with the various treatments. Calculations were normalized with GAPDH protein as a reference standard. **d** Snail expression is downregulated in the SiRNA control; however, in the TET2 and TET3 KDs, an increase in the expression of Snail in HLE and Huh7 was observed with the treatment. All the data are the average of the experiments (*N* = 3). **e** An increase in GADD45B expression was seen in the SiRNA controls treated with 5-AZA + vitamin C; however, within the TET2 and TET3 KDs, an increase in the expression of GADD45B expression was seen with the treatment with 5-AZA + vitamin C. Statistical significance was tested using one-way ANOVA (non-parametric),Tukey’s test. *p ≤ 0.05; **p ≤ 0.01; ***p ≤ 0.01. *Error bars* represent the standard deviation

Our results show that the HLE cells treated with vitamin C or 5-AZA individually show only small changes in the expression of mRNA and protein, while the combination of both substances results in a significant reduction of both Snail mRNA and protein levels (Fig. 4a, c).

In the Huh7 cells, a significant decrease in the mRNA was noted with vitamin C and 5-AZA independently and with a combination of 5-AZA + vitamin C while a corresponding decrease in protein was observed only with 5-AZA and the combination of 5-AZA + vitamin C (Fig. 4a, c).

We next studied the expression of the DNA damage-inducible gene GADD45B which is involved in G1 or G2 cell cycle arrest [31] and which has been implicated in/has been linked to the progression of HCC [32, 33]. We investigated whether the combined treatment of 5-AZA and vitamin C resulted in altered expression of GADD45B.

In both HLE and Huh7, an increase in GADD45B transcripts and proteins was observed with vitamin C and 5-AZA independently, but the increase was significant only with the combined treatment of 5-AZA + vitamin C (Fig. 4b, c). However, the combination of 5-AZA and vitamin C induced GADD45B mRNA and protein in both the HCC cell lines (Fig. 4b, c).

Further, to investigate whether the observed changes in the expression of Snail and GADD45 are TET-dependent, quantification of Snail and GADD45B transcripts was done in TET2 and TET3 KD of the HLE and Huh7 cells treated with 5-AZA + vitamin C. While Snail expression is downregulated in the SiRNA control, in the TET2 and TET3 KDs, an increase in the expression of Snail in HLE and Huh7 was observed with the treatment (Fig. 4d). Similar results were observed with GADD45B, where an increase in GADD45B expression was seen in the SiRNA controls treated with 5-AZA + vitamin C, however, within the TET2 and TET3 KDs, an increase in the expression of GADD45B was seen with the treatment of 5-AZA + vitamin C (Fig. 4e).

### Upregulated p21 and downregulated cyclin B1 expression induce cell cycle arrest

**Fig. 5:**
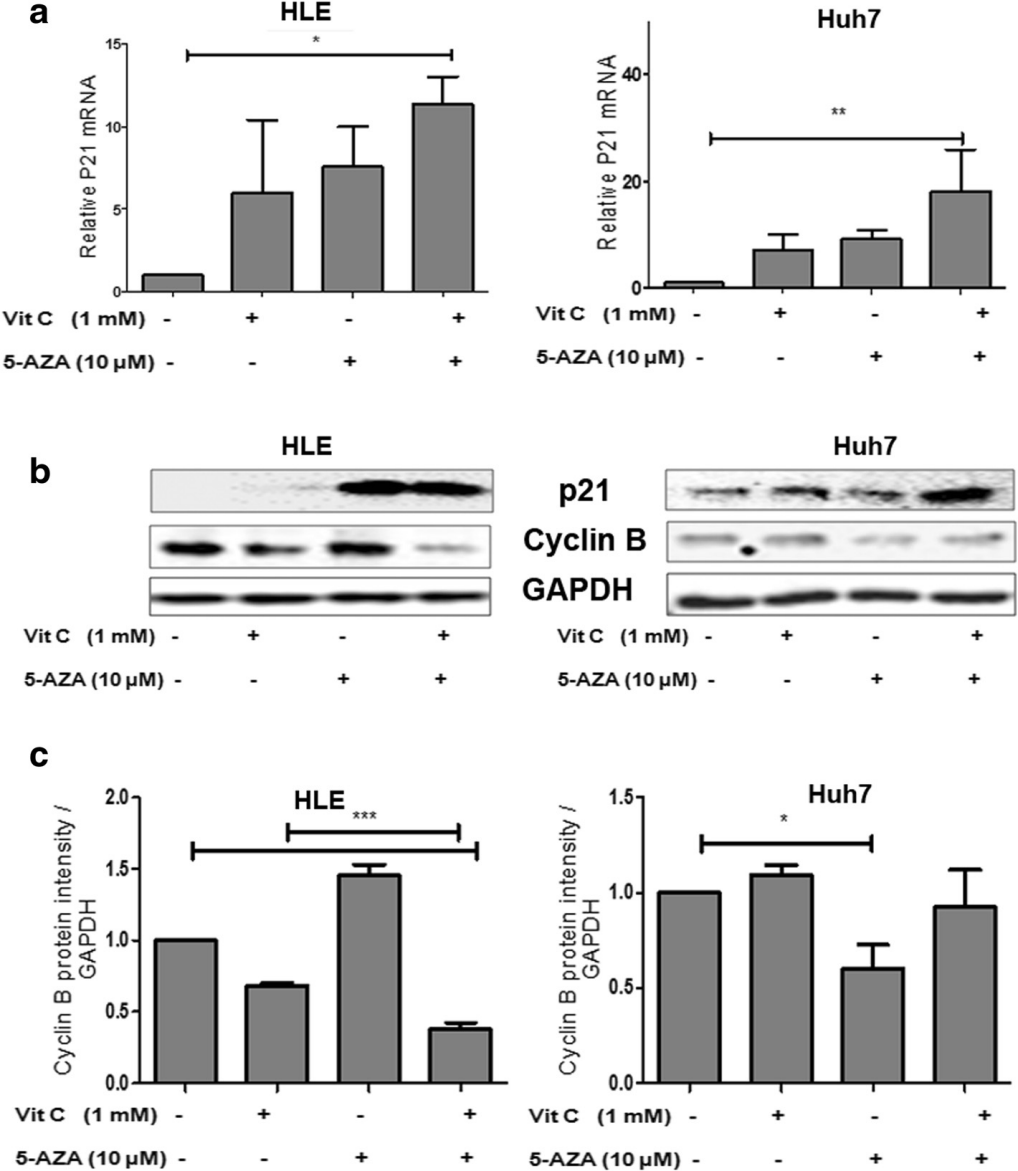
Increased P21 and reduced cyclin B1 by 5-AZA and vitamin C in HCC lead to cell cycle arrest. **a** Quantitative changes in P21 mRNA in HLE and Huh7 treated with vitamin C, 5-AZA, and 5-AZA + vitamin C compared to the untreated control after 48 h. Data was normalized using GAPDH expression as a reference control. **b** Western blot analysis for changes in P21 and cyclin B1 proteins in HLE and Huh7 with various treatments. Calculations were normalized with GAPDH protein. **c** Graphs represent the calculation of changes in cyclin B1 protein in HLE and Huh7 with the various treatments. Data was normalized using GAPDH expression as a reference control. All the data are the average of the experiments (*N* = 3). Statistical significance was tested using one-way ANOVA (non-parametric) Tukey’s test. *p ≤ 0.05; **p ≤ 0.01; ***p ≤ 0.01. *Error bars* represent the standard deviation

In the HLE and Huh7 cells, a significant upregulation of P21 mRNA was noted by the combination of 5-AZA + vitamin C as compared to 5-AZA or vitamin C individual treatments (Fig. 5a, b). In HLE, 5-AZA and 5-AZA + vitamin C treatments induced a significant increase in p21 protein, whereas in Huh7, the increase in p21 protein was higher in the combination treatment of 5-AZA + vitamin C than with 5-AZA alone. Since Snail represses p21 [34], it is possible that the increase in p21 may be attributed to a corresponding decrease in the Snail protein in both HLE and Huh7 leading to the arrest of the cells in G1, S, and or G2 phases of the cell cycle.

We also investigated changes in cyclin B1 protein expression as a downstream target of P21, which is essential for the progression of cells from G2 to the M phase of the cell cycle (Fig. 5b, c). In HLE, we observed that the expression of cyclin B1 decreased with independent vitamin C treatment but increased with independent 5-AZA treatment. However again, only the combination of 5-AZA + vitamin C reduced the expression of cyclin B1 significantly. As a consequence of cyclin B1 decrease, cells in the G2 phase are inhibited to progress to the M phase, which means that cell arrest takes place in the G2/M phase of the cell cycle. In the Huh7 cell line, however, we observed a significant decrease in cyclin B1 only with 5-AZA treatment.

### Increased E-cadherin expression indicates a possible shift of cells towards epithelial phenotype

**Fig. 6:**
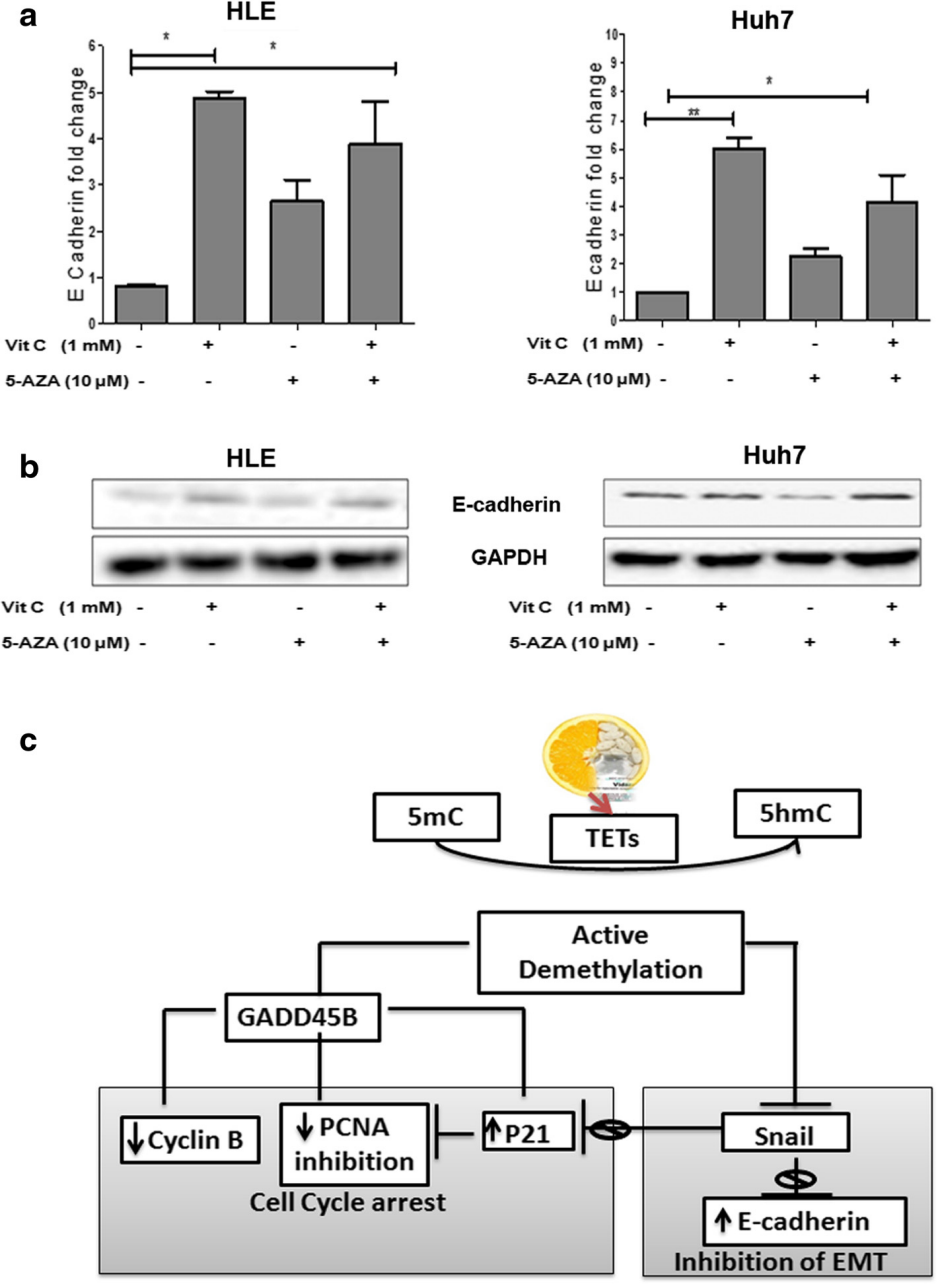
Increased E-cadherin expression in HCC treated with 5-AZA and vitamin C. **a** Quantitative changes in E-cadherin mRNA in HLE and Huh7 treated with vitamin C, 5-AZA, and 5-AZA + vitamin C compared to the untreated control after 48 h. Data was normalized using GAPDH expression as a reference control. **b** Western blot analysis for changes in P21 and cyclin B1 proteins in HLE and Huh7 with various treatments. Calculations were normalized with GAPDH protein. All the data are the average of the experiments (*N* = 3). Statistical significance was tested using one-way ANOVA (non-parametric) Tukey’s test. *p ≤ 0.05; **p ≤ 0.01; ***p ≤ 0.01). *Error bars* represent the standard deviation. **c** Vitamin C enhances active demethylation induced by 5-AZA, in converting 5-methyl cytosine to 5-hydroxymethyl cytosine by increasing the expression of TET2 and TET3. Induced demethylation leads to decrease in Snail expression and upregulation of GADD45B. Reduced Snail expression leads to upregulation of P21. The network of interactions between GADD45, P21, cyclin B1, and PCNA leads to the arrest of cells in G1, S, or G2/M phases of the cell cycle. On the other hand, increased expression of E-cadherin by reduction of Snail leads to the inhibition of EMT

Snail is a direct repressor of E-cadherin and induces EMT in HCC [33, 34]. In addition, it was reported that vitamin C induces MET by induction of TET proteins which may prevent cancer cells from attaining further invasive traits [11]. Thus, we were interested whether or not vitamin C and/or 5-AZA influence E-cadherin expression in HCC lines. In both HLE and Huh7 cell lines treated with vitamin C, 5-AZA, and 5-AZA + vitamin C, we observed an increase in E-cadherin mRNA and protein expression as compared to the untreated controls (Fig. 6a, b). In both HCC cell lines, the combination of 5-AZA + vitamin C showed a higher expression than 5-AZA alone. Further, it was interesting to note that in both cases, vitamin C independently was also able to induce a high expression of E-cadherin (Fig. 6a, b).

## Discussion

In this study, we have shown that vitamin C enhances the demethylation efficacy of 5-AZA by increasing the expression of TET2 and TET3 in HCC. In line with these data, we observed a significant cytotoxic increase when HCC cells were co-incubated with 5-AZA and vitamin C which is paralleled by an increased inhibition of cell proliferation, a decreased Snail expression, and an upregulation of the cell division kinase 2 (CDK-2) inhibitor; P21. The enhanced expression of GADD45B combined with the changes in expression of cyclin B1 and PCNA induced cell cycle arrest in HCC (Fig. 6c).

Recently, we have shown a decrease in cell viability and an increased cell damage in HCC cell lines treated with 5-AZA in a dose-dependent manner. Furthermore, we observed a significant induction of TET2 and TET3 at a concentration of 20 μM of 5-AZA [8]. It is interesting to note that the combination of 10 μM 5-AZA with vitamin C showed the same degree of epigenetic changes (5hmC intensity and TETs expression) like 20 μM 5-AZA. In the present study, we clearly demonstrated that vitamin C is able to enhance the epigenetic activity of 5-AZA, thus compensating for a higher dose of 5-AZA, necessary to induce cell cycle arrest in HCC.

Dynamic epigenetic changes mark EMT by the inducible expression of Snail [35]. Snail governs cell cycle progression by repressing P21 [36]. While Snail expression is essential for tumorigenesis, reduction in Snail expression in cancer cells is considered essential in limiting tumor cell progression by inducing cell cycle arrest [20, 21]. A recent study reports that 5-AZA inhibited the inducible Snail expression in cultured hepatocytes and also suggests the possible involvement of miR29b in Snail regulation [37]. Based on these studies and our recent report [8], we propose that downregulation in Snail expression is mainly attributed to an increase in TET activity by a combination of 5-AZA and vitamin C. Further, the combination of 5-AZA + vitamin C induced an increase in Snail expression in TET2 and TET3 KD cells of both HLE and Huh7 in contrast to the decrease in expression which was seen when TET2 and TET3 were intact. These results further emphasized that Snail expression was influenced by increased TET2 and/or TET3 activity upon treatment with 5-AZA and vitamin C. It is reported that miRNAs play an important role in limiting Snail expression [37]. The decrease in Snail expression with the treatment, despite the corresponding increase in TETs suggests that the effect of TETs on Snail is indirect, with the probable TET mediated upregulation of miRs or other repressors of Snail upstream of the pathway playing a significant role in limiting Snail expression. The details of this mechanism, however, are yet to be elucidated.

The growth arrest and DNA damage-inducible GADD45 genes are central players that are upregulated during cellular stress. Activation of GADD45 results in several processes of growth arrest, DNA repair, survival, or apoptosis (reviewed in [38]). Hypermethylation of the GADD45 promoter is found in various cancers including HCCs, which leads to downregulation of GADD45 expression and promotes tumor progression [32, 38]. It was reported that 5-AZA enhances the expression of GADD45 in colon cancer cells resulting in the induction of apoptosis [22]. GADD45 proteins are also reported to induce cell cycle arrest by direct interaction with P21, PCNA, and cyclin B1 (through CDK1/cyclin B1 complex) thereby inducing cell cycle arrest at the various phases of the cell cycle [39–41]. P21 is also known to have a PCNA-binding domain towards its carboxyl terminal and to inhibit the replication of DNA at the S phase [42]. In the same line of evidence, it was also reported that binding of P21 to PCNA induces cell cycle arrest at the G1 or G2 level [43]. In agreement with the above reports, we have seen an enhanced expression of GADD45 in our HCC cells treated with a combination of 5-AZA and vitamin C. It has been reported recently that GADD45 could also induce active demethylation processes via nucleotide excision repair or base excision repair pathway [44] which might also be associated with the induction of active demethylation via 5-AZA and vitamin C, as shown in this study.

The most common problem associated with tumor cells is their increased resistance to programmed cell death. Induction of E-cadherin expression is considered an important step in sensitizing tumor cells towards apoptosis [45]. E-cadherin modulates apoptosis by coupling with the death receptors DR4/DR5. Consequently, increased expression of E-cadherin sensitizes cancerous cells to cell death [46]. Therefore, reactivation of E-cadherin could be an important target for epigenetic therapy in HCC. 5-AZA, when used either alone or in combination with other drugs, was shown to enhance the expression of E-cadherin in HCC cell lines and lung epithelial cells, respectively [45]. In differentiated hepatocytes, 5-AZA not only maintains the expression but also inhibits downregulation of E-cadherin [37]. In the present study, since Snail is a direct repressor of E-cadherin [16], reduction of Snail was expected to have an enhanced expression of E-cadherin. In line with this, we observed an increase in the expression of E-cadherin by a combination of 5-AZA + vitamin C. An increase in the number of cells undergoing cell death with the combined treatment is probably due to both the arrest of cells by reduced Snail and the increased sensitivity of the cells towards cell death by the enhanced expression of E-cadherin.

An increase in E-cadherin expression upon independent treatment with vitamin C further highlights the role of vitamin C in epigenetic regulation. The use of vitamin C could therefore support the maintenance of epithelial morphology of the cells and thus prevent the MET, which is involved in cancer cell invasion and progression [47].

## Conclusions

Epigenetics plays a crucial role in tumorigenesis by modulating EMT and cell cycle proliferation pathways. We have shown that vitamin C enhances the demethylation activity of the epigenetic drug 5-AZA and induces cell cytotoxicity. Inhibition of Snail expression leads to upregulation of p21 and simultaneous activation of GADD45 which are considered as the major effectors in inducing cell cycle arrest, as shown in the HCC cell lines treated with 5-AZA and vitamin C (Fig. 6c). Our study has added value to the growing evidences of vitamin C as an epigenetic player. Though further studies are yet warranted, our results suggest the possible exploration of the use of epigenetic drugs in combination therapies as an attractive future strategy for cancer treatment.

## Abbreviations

5-AZA: 5-azacytidine
5hmC: 5-hydroxymethylcytosine
5mC: 5-methylcytosine
CDK-2: cell division kinase 2
DNMTi: DNA methyl transferase inhibitor
EMT: epithelial-mesenchymal transition
FC: flow cytometry
HCC: hepatocellular carcinoma
KD: knock-down
LDH: lactose dehydrogenase
MET: mesenchymal-epithelial transition
PCNA: proliferating cell nuclear antigen
PI: propidium iodide
TET: ten-eleven translocation
